# Caprin-1 plays a role in cell proliferation and Warburg metabolism of esophageal carcinoma by regulating METTL3 and WTAP

**DOI:** 10.1186/s12967-023-04001-0

**Published:** 2023-02-28

**Authors:** Yan Gao, Lingling Yuan, Changbin Ke, Zhijun Pei, Xiaobo Liu, Ruimin Wu, Xueyan Kui, Yanmin Zhang

**Affiliations:** 1 School of Pharmacy, Health Science Center, Xi’an Jiaotong University, Xi’an, 710061 People’s Republic of China; 2 Department of Nuclear Medicine and Institute of Anesthesiology and Pain, Department of Pathology, Taihe Hospital, Hubei University of Medicine, Shiyan, 442000 People’s Republic of China

**Keywords:** Caprin-1, Esophageal carcinoma, ^18^F-FDG-PET, Glucose metabolism, Methyltransferase-like 3, Wilms’ tumor 1-associating protein

## Abstract

**Background:**

Cytoplasmic activation/proliferation-associated protein-1 (Caprin-1) is implicated in cancer cell proliferation and tumorigenesis; however, its role in the development of esophageal carcinoma (ESCA) has not been examined.

**Methods:**

Biological methods and data analysis were used to investigate the expression of Caprin-1 in ESCA tissue and cell lines. We comprehensively analyzed the mRNA expression and prognostic values, signalling pathways of CAPRIN1 in ESCA using public databases online. Biological functions of CAPRIN1 were performed by clorimetric growth assay, *EdU* staining, colony formation, flow cytometry, apoptosis analysis, Western blot, lactate detection assay, extracellular acidification rates. The underlying mechanism was determined via flow cytometric analysis, Western blot and rescue experiments. In addition, xenograft tumor model was constructed to verify the phenotypes upon CAPRIN1 silencing.

**Results:**

Caprin-1 expression was significantly elevated in both ESCA tumor tissues and cell lines compared with that in normal adjacent tissues and fibroblasts. Increased CAPRIN1 mRNA expression was significantly associated with clinical prognosis and diagnostic accuracy. The GO enrichment and KEGG pathway analysis CAPRIN1 might be related to immune-related terms, protein binding processes, and metabolic pathways. A significant positive correlation was observed between high Caprin-1 protein levels and lymph node metastasis (*P* = 0.031), ki-67 (*P* = 0.023), and ^18^F- FDG PET/CT parameters (SUVmax (*P* = 0.002) and SUV mean (*P* = 0.005)) in 55 ESCA patients. At cut-off values of SUVmax 17.71 and SUVmean 10.14, ^18^F- FDG PET/CT imaging predicted Caprin-1 expression in ESCA samples with 70.8% sensitivity and 77.4% specificity. In vitro and in vivo assays showed that Caprin-1 knockdown affected ESCA tumor growth. Silencing Caprin-1 inhibited ESCA cell proliferation and glycolysis, and decreased the expression of methyltransferase-like 3 (METTL3) and Wilms’ tumor 1-associating protein (WTAP). However, this effect could be partially reversed by the restoration of METTL3 and WTAP expression.

**Conclusions:**

Our data suggest that Caprin-1 could serve as a prognostic biomarker and has an oncogenic role in ESCA.

**Supplementary Information:**

The online version contains supplementary material available at 10.1186/s12967-023-04001-0.

## Background

Esophageal carcinoma (ESCA) is a common malignant cancer worldwide, especially in China [1], and has two main histological subtypes: esophageal squamous cell carcinoma (ESCC) (accounting for 90% of total ESCA cases) and esophageal adenocarcinoma (EA). Despite significant advances in ESCA diagnosis, prognosis, and treatment, the 5-year survival rate of patients remains unsatisfactory [2]. Therefore, new biological or pathological biomarkers and disease-specific molecular mechanisms underlying ESCA progression are urgently needed. Bioinformatic analysis and experimental approaches have been combined to explore novel predictive and prognostic markers for diverse cancers [3, 4].

The Warburg effect, also known as aerobic glycolysis, is metabolic hallmark of cancer cells [5]. Positron emission tomography/computed tomography (PET/CT), using a glucose analogue named ^18^F-fludeoxyglucose (^18^F-FDG), could reflect the glycolysis levels of tumors and has been widely used for early diagnosis, staging, and treatment assessment of ESCA [6, 7]. The ^18^F-FDG uptake rate was quantified by PET metabolic parameters like the maximum and mean standardized uptake value (SUVmax, SUVmean), metabolic tumor volume (MTV), and total lesion glycolysis (TLG). Tumour standardised uptake value (SUV) has been shown to be a useful PET metabolic parameter for risk assessment in cancers. PET metabolic parameters have been reported to be significantly associated with prognostic biomarkers expression in different tumors both in vitro and in vivo [8–11].

Cytoplasmic activation/proliferation-associated protein-1 (Caprin-1) [12], is involved in neurodegenerative diseases [13, 14] and various cancers [15–18]. Caprin-1 acts as RNA binding protein and participates in extensive biological and physiological processes, such as cell proliferation, RNA modification [19], and immune response [20]. N6-methylandenosine (m6A) is the most abundant modification in mammalian mRNA, and it plays important roles in cancer development [21]. Integrative network analysis has identified Caprin-1 as m6A regulator that selectively promotes methylations of m6A sites through physical interactions with m6A writers, such as methyltransferase-like 3 (METTL3) and methyltransferase-like 14 (METTL14) [19]. METTL3, METTL14, and Wilms’ tumor 1-associating protein (WTAP) were identified as components of the human m6A methyltransferase complex, and WTAP interacts with METTL3 and METTL14 [22, 23]. However, disease-relevant expression profiles and biological functions of Caprin-1, and its correlations with the hallmark of ESCA have not yet been elucidated.

In the present study, we investigated the relationship between the expression and the prognosis value of Caprin-1, as well as its potential mechanisms of action in ESCA, through a comprehensively bioinformatics analysis of data from The Cancer Genome Atlas (TCGA) [4] and Gene Expression Omnibus (GEO) [24]. The experimental methods and correlation analysis of Caprin-1 with ^18^F-FDG PET parameters in ESCA were combined to explore mechanisms underlying the functions of Caprin-1 in tumor growth and glycolysis reprogramming. Our results indicate that Caprin-1 could be a novel diagnostic and prognostic biomarker for patients with ESCA.

## Methods

### Patient samples

The study respectively reviewed data from 55 patients who were surgically treated and had pathologically confirmed ESCC in Taihe Hospital from January 2018 to July 2020. All patients met the following inclusion criteria: (a) they underwent ^18^F-FDG PET/CT imaging prior to chemotherapy or radiation therapy; (b) their tissue samples were available for immunohistochemistry (IHC) staining; and (c) their complete demographic information, including gender, age, tumor size, pathological type, histological differentiation, lymph node metastasis, and pathological stage ( p stage), was available. Another 14 paired samples, including tumor tissues and adjacent normal tissues from patients with ESCC were collected for Western blot experiments.

### ^18^F-FDG PET/CT imaging and data analysis

Glucose metabolism imaging was performed on a ^18^F-FDG PET/CT system (Biograph mCT-64; Siemens Healthcare, Erlangen, Germany). As described previously [10, 11], all patients fasted for at least 6 h, but had free access to water until the start of imaging. Whole-body position was monitored 50 min after intravenous FDG administration with a dose of 3.7–4.1 MBq/kg and lasted for approximately 15 min. PET/CT images were obtained according to the manufacturer’s protocol [23]. Metabolic parameters of ^18^F-FDG PET images were visually evaluated by two experienced nuclear medicine physicians blinded to the final clinical diagnosis. Briefly, a region of interest was placed around the primary tumor; FDG uptake in lesions was determined for calculation of PET parameters.

### IHC and immunofluorescence stain assay

IHC staining of esophagus tissue was employed to investigate protein expression as previously described [10, 11]. Samples were dissected into 5-μm-thick tissue sections embedded in paraffin, and incubated with antibodies against Caprin-1 (ProteinTech Group, Chicago, IL, USA), and Ki-67 (Zhongshan Golden Bridge Biotechnology, Beijing, China). All data were analyzed by two independent pathologists blinded to patient information. For Ki-67 evaluation, we considered the percentage of nuclear staining for scoring proliferative status. Ki-67 proliferation index ≥ 10% was defined as Ki-67 positive. Ki-67/Caprin-1 protein expression was determined according to the staining intensity score: 0 (no staining), 1 (weak staining, light yellow), 2 (moderate staining, yellow brown), and 3 (strong staining, brown). Final intensity scores of < 2 and ≥ 2 were considered low and high expression, respectively. The clinical patients were divided into Caprin-1/Ki-67 low and high groups based on their IHC paraffin staining score ranking.

For immunofluorescence staining, cultured cells on slides were washed, fixed and stained with Caprin-1 primary antibody and with Donkey anti-goat Alexa Fluro 488 (Abcam, Cambridge, England) as previously reported [25, 26]. Next, slides were mounted with DAPI (Thermo Fisher Scientific, Waltham, MA, USA) to stain cell nuclei and observed under a confocal fluorescence microscope (LEICA TCS SP8; Leica Microsystems, Wetzlar, Germany).

### Gene expression pattern and survival prognosis analysis in public datasets

The mRNA expression profiles (HTSeq counts) and associated clinical data of the CAPRIN1 in ESCA patients and cell lines were obtained from the TCGA website and the GEO database (GSE63941, GSE69925). Expression profiles were plotted in the ggplot2 R. The TIMER database [27] was also used for mRNA expression analysis. Survival curve analysis was performed to assess the correlation between the expression of different genes and survival rates, in the Kaplan–Meier plots database [28]. Area under curve was used for analysis of prognostic or predictive accuracy. The “pROC” package in R was used to generate the receiver operating characteristic (ROC) curve, which allowed for time-dependent analysis with the censored data of TCGA cancers.

### Function and network analysis using Cytoscape and gene-set enrichment analysis (GSEA)

The co-expression genes of CAPRIN1 in ESCA were downloaded from LinkedOmics database, wherein multi-omics data were analyzed across 32 TCGA cancer types [29]. Subsequently, gene ontology (GO) data for BP (biological process), CC (cellular component), and MF (molecular function) of co-expressed genes were investigated. Next, the top 100 correlated genes were screened out for the Protein-protein interaction (PPI) networks. PPI data were obtained from the String database (https://string-db.org/, version 11.5, updated on August 12, 2021). The top 20 hub genes were identified by degree using a plugin named cytoHubba in Cytoscape.

GSEA was also performed using the GEO matrix of the GSE69925 cohort to investigate the potential role of CAPRIN1 in the high- and low-expression groups based on C5 (Gene Ontology, GO) and C2 [Canonical pathways, Kyoto Encyclopedia of Genes and Genomes (KEGG)]. Investigated glycolysis signatures included REACTOME_GLYCOLYSIS, HALLMARK_GLYCOLYSIS, KEGG_GLYCOLYSIS, and the MOOTHA_GLYCOLYSIS. These gene sets were downloaded from the Molecular Signatures Database (MSigDB). False discovery rate < 0.25 and nominal *P* < 0.05 denoted the cutoff for significance.

### Cell culture and treatment

Human ESCC cell lines Eca109 and KYSE150 were the kind gift of Prof. Luo (Hubei University of Medicine, China) and were mycoplasma-free. Eca109 cells were cultured in DMEM (Hyclone Laboratories Inc, Logan, UT, USA), and KYSE150 cells were maintained in RPMI 1640 (Hyclone Laboratories Inc). The medium was supplemented with 10% fetal bovine serum (Thermo Fisher Scientific). Small interfering RNA (siRNA, 100 pmol) against human CAPRIN1 was transfected into cells with Lipofectamine 8000 transfection reagent (Beyotime Biotechnology, China), while a non-specific siRNA was used as a negative control. The siRNA sequences were as follows: Caprin-1 siRNA 1# (siCaprin-1 1#), sense 5′-CCAGGAAGUCACAAAUA AUTT-3′; antisense 5′-AUUAUUUGUGACUUCCUGGTT-3′; Caprin-1 siRNA 2# (siCaprin-1 2#), sense 5′-GGGACCUGCUGGAAGGGAATT-3′; antisense 5′-UUCCCUUCCAGCAGGUCCCTT-3′; siNC, sense 5′-ACGUGACACGUUCGGAG AA-3′; antisense 5′-UCUUCUCCGAACGUGUCACGU-3′. Eca109 cells were transfected with Caprin-1 knockdown lentivirus (termed as shCaprin-1) to select stably transfected hybrid colonies; and transfected with plasmid overexpressing METTL3 or WTAP (METTL3 OE or WTAP OE) generated by Genechem.

### Real-time quantitative PCR and Western blot

For mRNA analysis, total RNA extraction for cDNA synthesis was conducted with the Taqman Reverse Transcription Reagent kit (TaKara-Bio, Kusatsu, Japan). Real-time quantitative PCR was conducted with different primer sequences (Table 1) using SYBR Green Master Mix (TaKaRa).

**Table 1:** Primer sequences for real time RT-PCR

Target	Forward	Reverse
ACTB	TCTTCCAGCCTTCCTTCCT	AGCACTGTGTTGGCGTACAG
CAPRIN1	TCTCGGGGTGATCGACAAGAA	CCCTTTGTTCATTCGTTCCTGG
SLC2A1	TGTCTGGCATCAACGCTGTCTTC	TC CCTGCTCGCTCCACCACAAAC
HK2	CGACAGCATCATTGTTAAGGAG	CA GCAGGAAAGACACATCACATTT
HIF1A	AGTTCCGCAAGCCCTGAAAGC	GCAGTGGTAGTGGTGGCATTAGC
MYC	CGCCTCTTGACATTCTCCTC	GGACTATCCTGCTGCCAAGA
NUP160	GTTATCTGGCTGCTCTCAATTG	GTGCATTCTCCATCATGATTCC
NUP133	AGTACCTGTGGGCTGCTTCTCTAG	GCTCTGGTTGTCAGTCTGCTCAC
NUP155	CCGCTCCTCAGTCTCCCAGTG	GCTCATCCTTGGATCGCTGTGAC
METTL3	CCAGCACAGCTTCAGCAGTTCC	GCGTGGAGATGGCAAGACAGATG
WTAP	CTGACAAACGGACCAAGTAATG	AAAGTCATCTTCGGTTGTGTTG

Proteins were separated by SDS–PAGE followed by Western blot as described previously [30, 31]. Anti- GAPDH (Cell Signaling Technology), anti-Caprin-1 (116 kDa, ProteinTech Group), anti-HIF1α (120 kDa, ProteinTech Group), anti-c-Myc (49 kDa, roteinTech Group), anti-WTAP (45 kDa, Santa Cruz Biotechnology), and anti-METTL3 (64 kDa, Abcam) were used as primary antibodies. HRP-conjugated anti-mouse and anti-rabbit IgG (Cell Signaling Technology) were used as secondary antibodies.

### Cell proliferation assay

Cells were maintained in 96-well plates (5 × 10^3^ cells/well) for 24 h and transfected with siRNA. Cell viability and growth rate were assessed using CellTiter 96 AQueous One Solution Cell Proliferation Assay (MTS) kit (Promega, Madison, WI, USA) following the manufacturer’s instructions. Cell proliferation was assessed using the BeyoClick™ *EdU*-488 *assay* kit (Beyotime). Briefly, 3 × 10^4^ cells were seeded in 24-well cover-glass bottom plates, incubated for 24 h, and transfected with siRNA. Then the cells were treated with 10 μM Edu for 2 h, fixed, permeabilized, and stained with the Click Additive Solution. Cell nuclei were stained with DAPI. Images were captured using a Leica SP8 laser-scanning confocal microscope. The cell proliferation rate was calculated as a percentage of EdU-positive cells per DAPI-positive cells. For the colony formation assay, cells were seeded into 12-well plates and treated with siRNA. After 14 days, visibly stained colonies were counted using Image J software.

### Apoptosis detection

Cell apoptosis was assessed by flow cytometry (BD FACS Vantage SE, Franklin Lakes, NJ, United States) using Apoptosis Detection Kit I (BD Biosciences Pharmagen, San Diego, CA, USA) according to the manufacturer’s instructions. Treated cells binding only to Annexin V were classified as early apoptotic, while double-stained cells were classified as late apoptotic [31].

### Lactate detection assay

To measure lactate production, Eca109 cells were seeded in 96-well plates (8 × 10^3^ cells/well) in triplicate. The culture medium was collected to measure lactate concentration as determined by a lactate assay kit (Eton Bioscience Inc, San Diego, CA, USA) 48 h after siRNA transfection.

### Extracellular acidification rates (ECARs)

ECARs were measured using an XF24 extracellular analyzer (Seahorse Bioscience, North Billerica, MA, USA) [32]. Briefly, 20,000 Eca109 cells/well were cultured in the XF24 cell culture plate with medium at 48 h after siRNA transfection. Cells were washed with PBS, the respective XF assay medium with 2 mM glutamine was added to each well, and the plate was incubated at 37 °C for approximately 45 min. After analyzer calibration, sequential compound injections, including glucose, oligomycin A and 2-DG (AmyJet Scientific, Wuhan, China), were applied to test glycolytic activity.

### Animal model

For the in vivo xenograft implantation assay, male BALB/c athymic nude mice (five mice per group) were subcutaneously injected with stable Caprin-1 knockdown Eca109 cells. Tumor volumes were measured twice every week, and calculated using the following formula: volume (mm^3^) = longest diameter × shortest diameter^2^/2. After 5 weeks, animals were sacrificed and tumors were removed for further examination.

### Statistical analysis

Statistical analysis were conducted using SPSS Statistics for Windows, version 16.0 (SPSS Inc., Chicago, IL, USA) and R package version (4.0.3). Significance levels were set at *P* < 0.05. Survival and ROC analyses were performed in R or in the corresponding R packages survival; survminer, and pROC. Unpaired two-tailed Student’s t tests were applied to compare data between two groups, and one-way ANOVA was used for multiple comparisons (CAPRIN1 expression in patient with differing race and histological types, in vitro and in vivo expiments). Proportional differences in clinicopathological characteristics were analyzed by chi-squared. For comparisons of medians in groups (PET metabolic parameter TLG and MTV), nonparametric statistical tests (Kruskall-Wallis test) were used. Spearman’s rho test was used to evaluate correlations between PET parameters and CAPRIN1 expression in ESCA patients. Multivariate Cox regression analysis was applied to identify the predictor for positive Caprin-1 expression. The greatest Youden index (sensitivity + specificity –1) was used to depict the optimal cutoff value for the ROC curve. To investigate the correlation between the expression of Caprin-1 and m6A-related genes in the TCGA data set, 20 m6A-related genes [21] were derived from previous work. All aforementioned analyse and R packages were implemented using R Foundation for Statistical Computing (2020) version 4.0.3 and software packages, including ggplot2 pheatmap.

## Results

### Clinicopathological characteristics of Caprin-1 in ESCA patients

**Fig. 1:**
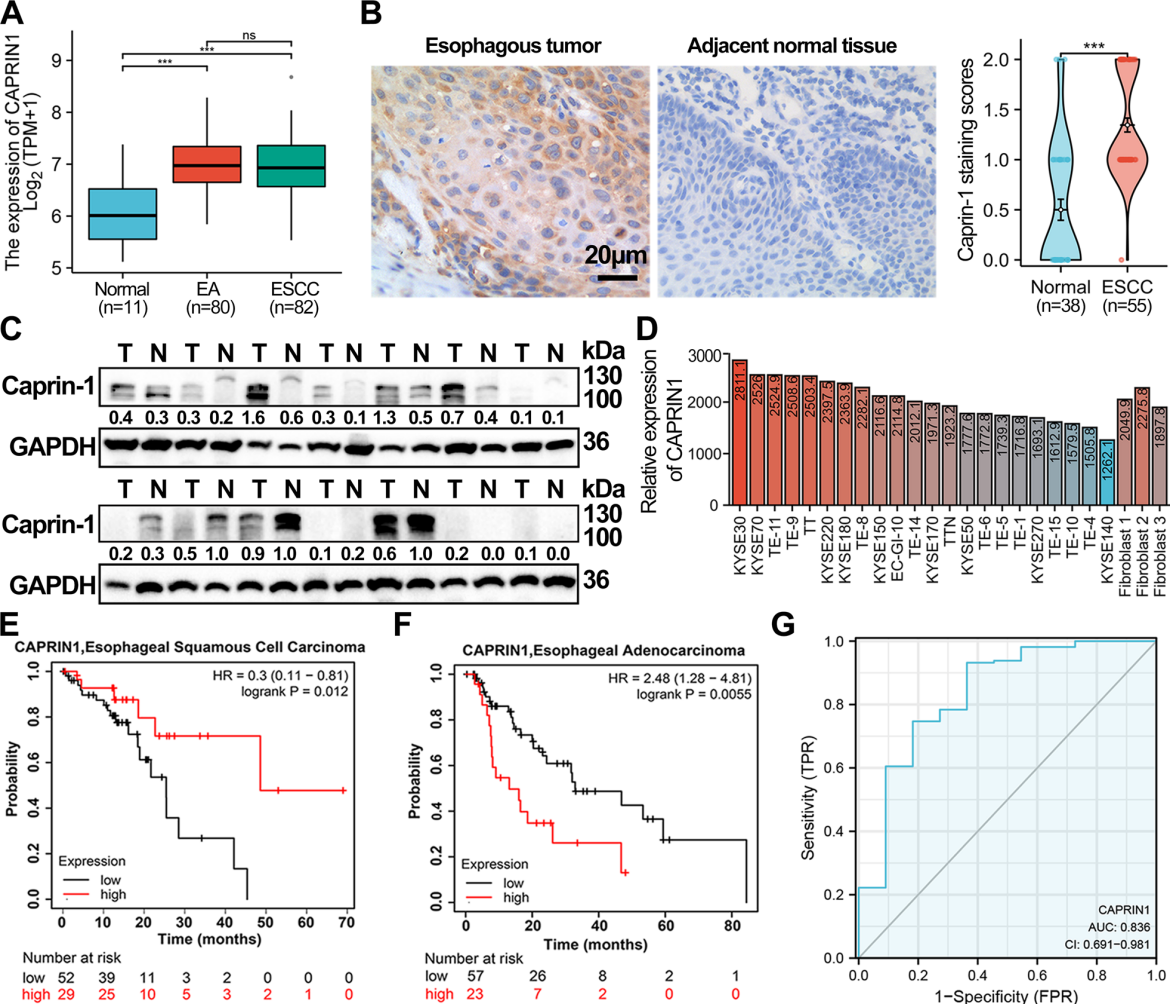
Expression pattern and overall survival (OS) rates of CAPRIN1 in ESCA patients and cell lines. **A** Comparison of CAPRIN1 expression among EA, ESCC, and normal tissues based on data from the TCGA-ESCA cohort. **B** Immunohistochemical staining and quantification of Caprin-1 protein in ESCC tissues and adjacent normal tissues. **C** The protein expression level of Caprin-1 in 14 paired ESCC tissues was examined using Western blot. T, tumor; N, adjacent normal tissues. **D** CAPRIN1 mRNA expression levels in different ESCA cell lines and fibroblast cultured from esophageal squamous cell carcinoma tissue in the GEO cohort (GSE63941). **E** OS analysis according to the mRNA expression of CAPRIN1 in ESCC and EA **F** patients using a Kaplan–Meier plotter. **G** The ROC curve of CAPRIN1 for the diagnosis of patients with ESCA. * P < 0.05, ** P < 0.01, *** P < 0.001

The mRNA and protein expression of CAPRIN1 was up-regulated in ESCA tissues than in normal tissues (*P* < 0.05; Fig. 1A and B). Similarly, Western blot analysis confirmed the results in the collected tissues (*P* < 0.05, Fig. 1C). We observed high CAPRIN1 levels in several ESCA cell lines, especially in KYSE30 and KYSE70, compared with fibroblast cultured from esophageal squamous cell carcinoma tissue using the GEO database (GSE63941, Fig. 1D).

Clinicopathological parameters of the 55 patients with ESCC are presented in Table 2. Caprin-1 expression in ESCC patients was significantly associated with lymph node metastasis (*P* = 0.031), Ki-67 expression (*P* = 0.023), SUVmax (*P* = 0.002), and SUVmean (*P* = 0.005), but not with gender, age, tumor size, differential status, p stage, TLG or MTV. But CAPRIN1 mRNA expression was significantly negatively associated with age (*P* < 0.05), not with races including in ESCA and ESCC tumors (Additional file 1: Fig. S1A and B). Caprin-1 expression was negatively associated with OS in 81 ESCC patients (HR = 0.30, 95% CI: 0.11–0.81, *P* = 0.012; Fig. 1E) and was significantly positively associated with OS in 80 EA patients (HR = 2.48, 95% CI: 1.28–4.81, *P* = 0.006; Fig. 1F) in TCGA database. Finally, CAPRIN1 showed high diagnostic value in ESCA (area under the curve = 0.836; Fig. 1G). Collectively, these results suggest that Caprin-1 was up-regulated in ESCA and could serve as a potential diagnostic indicator for patients with ESCA.

**Table 2:** Clinicopathological characteristics of 55 patients

Variables	N	Caprin-1- Low	Caprin-1- High	*P*-value
Total Clinical parameters	55	31	24	
Gender				0.099
Male	43	22	21	
Female	12	9	3	
Age (years)				0.210
< 60	32	20	12	
≥ 60	23	11	12	
Tumor size (cm)				0.199
≤ 3	31	15	8	
> 3	23	16	16	
Differential				0.424
Poorly	21	11	10	
High/Moderately	34	20	14	
Lymph node metastasis				0.031*
Negative	34	23	11	
Positive	21	8	13	
p Stage				0.102
1	28	19	9	
2	19	7	12	
3	8	5	3	
Ki-67 status				0.023*
Low	28	23	5	
High	27	14	13	
PET metabolic parameters				
SUVmax (median)				0.002*
Low	29	22	7	
High	26	9	17	
SUVmean (median)				0.005*
Low	28	21	7	
High	27	10	17	
TLG (median)				0.120
Low	29	19	10	
High	26	12	14	
MTV (median)				0.175
Low	28	18	10	
High	27	13	14	

*P < 0.05

### Immune-related terms and metabolic pathways significantly enriched for CAPRIN1 mRNA expression

**Fig. 2:**
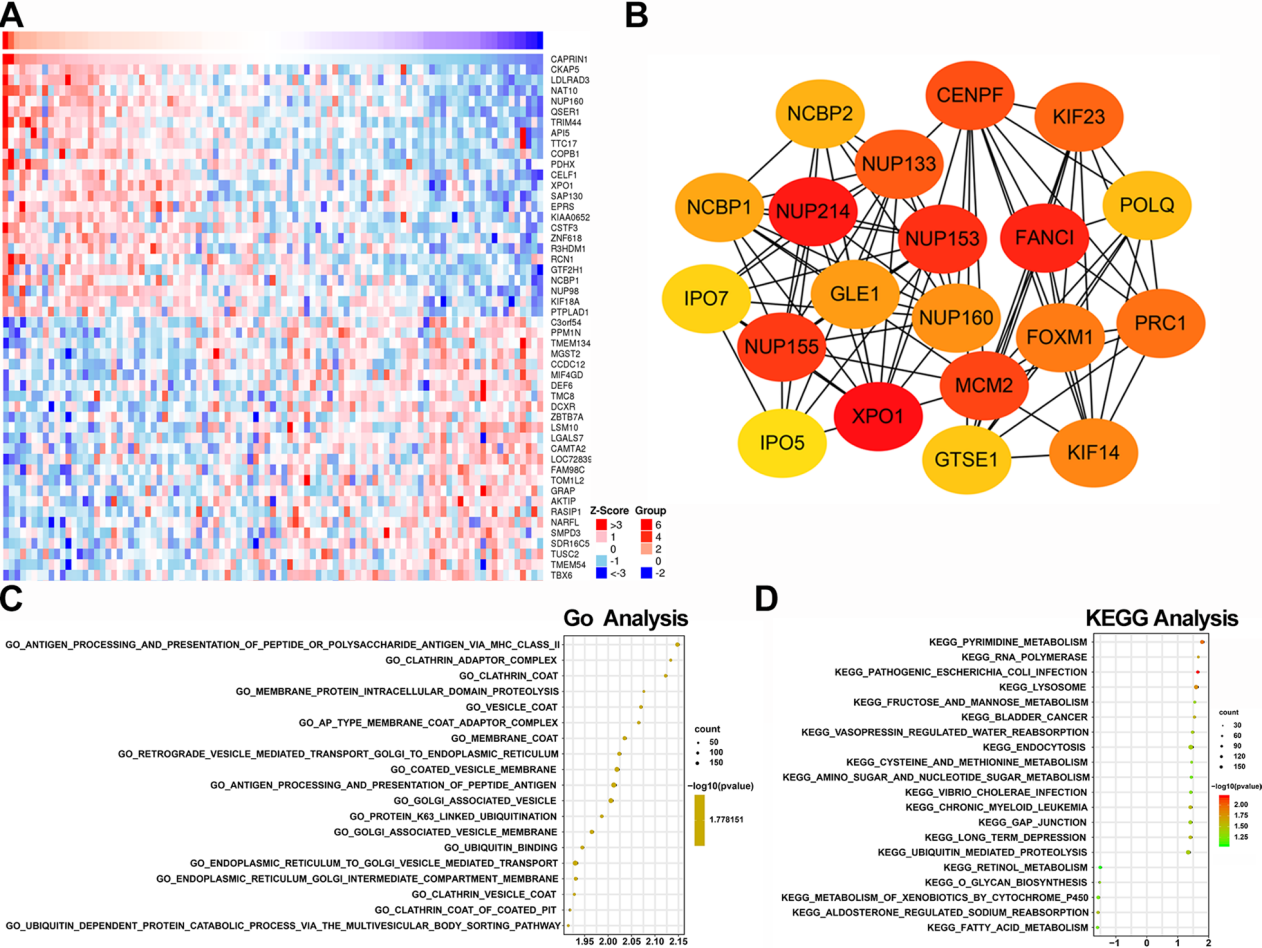
Differentially expressed genes and gene set enrichment analysis (GSEA) with high and low CAPRIN1 expression groups. **A** The heat map of the top 25 genes positively (red) and negatively (blue) correlated with CAPRIN1 in the TCGA-ESCA database. **B** The top 20 hub genes screened in the PPI network using the cytoHubba module of Cytoscape (higher color represents stronger connectivity). **C** The most significantly enriched GO annotations and KEGG pathways **D** of CAPRIN1 in the GEO database (GSE69925) by GSEA

To further explore the underlying mechanism, a group of genes correlated positively and negatively with CAPRIN1 expression were screened (Fig. 2A). The interactions between 156 genes are shown in Additional file 1: Fig. S1C. The top 20 hub genes were screened and ranked by the number of nodes, such as *XPO1, NUP214, NUP155, NUP153* (Fig. 2B). Differentially expressed genes were highly associated with metabolic process, membrane, and protein binding process (Additional file 1: Fig. S1D). Significant differences (false discovery rate < 0.25, NOM *P* -value < 0.05) in the enrichment of GO and KEGG collection in 85 ESCA patients (Fig. 2C, D). Thus, overall functions of DEGs seemed to map on immune-related GO terms, such as antigen processing and presentation of peptides or polysaccharide antigens via MHC class II, membrane, and protein binding processes such as clathrin adaptor complex and coat. The KEGG enrichment analysis indicated the most significantly enriched signaling pathways, such as primary metabolism, RNA polymerase and pathogenic *Escherichia coli* infection.

### Suppression of Caprin-1 inhibited ESCA cell proliferation by inhibiting glycolysis

**Fig. 3:**
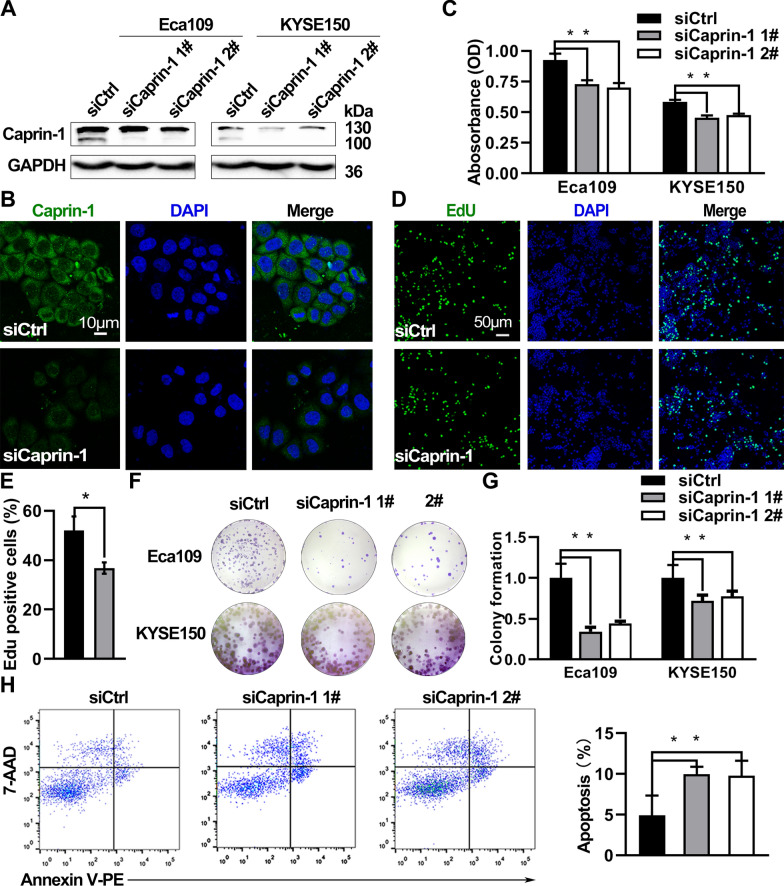
Down-regulation of Caprin-1 inhibited cell proliferation and induced apoptosis. **A** Western blot showing effective knockdown of Caprin-1 protein in two ESCA cell lines. **B** Immunofluorescence staining of Caprin-1 in Eca109 cells. **C** Colorimetric growth assay in Eca109 cells and KYSE150 cells transfected with Caprin-1 siRNAs or control oligos. **D** Representative images and quantification **E** of Edu positive rate in Eca109 cells. **F** Colony formation assay and quantification **G** in Eca109 cells and KYSE150 cells. **H** Flow cytometry analyses of cell apoptosis rate in Eca109 cells

Western blotting and immunofluorescence assay (Fig. 3A, B) revealed that both siRNA 1# and siRNA 2# resulted in a significant down-regulation of Caprin-1 protein expression in Eca109 and KYSE150 cells. Caprin-1 expression was primarily localized to the cytoplasm of Eca109 cells (Fig. 3B). Caprin-1 knockdown in Eca109 and KYSE150 cells suppressed cell growth (Fig. 3C). The EdU assay showed that Caprin-1 knockdown resulted in 30% decreased proliferation in Eca109 cells (Fig. 3D, E). Colony formation assay revealed that Caprin-1 siRNA inhibited colony formation potential of both cells (Fig. 3F, G). Caprin-1 siRNA induced early apoptosis of Eca109 cells (Fig. 3H). These results indicated that Caprin-1 knockdown could inhibit ESCA cell proliferation.

**Fig. 4:**
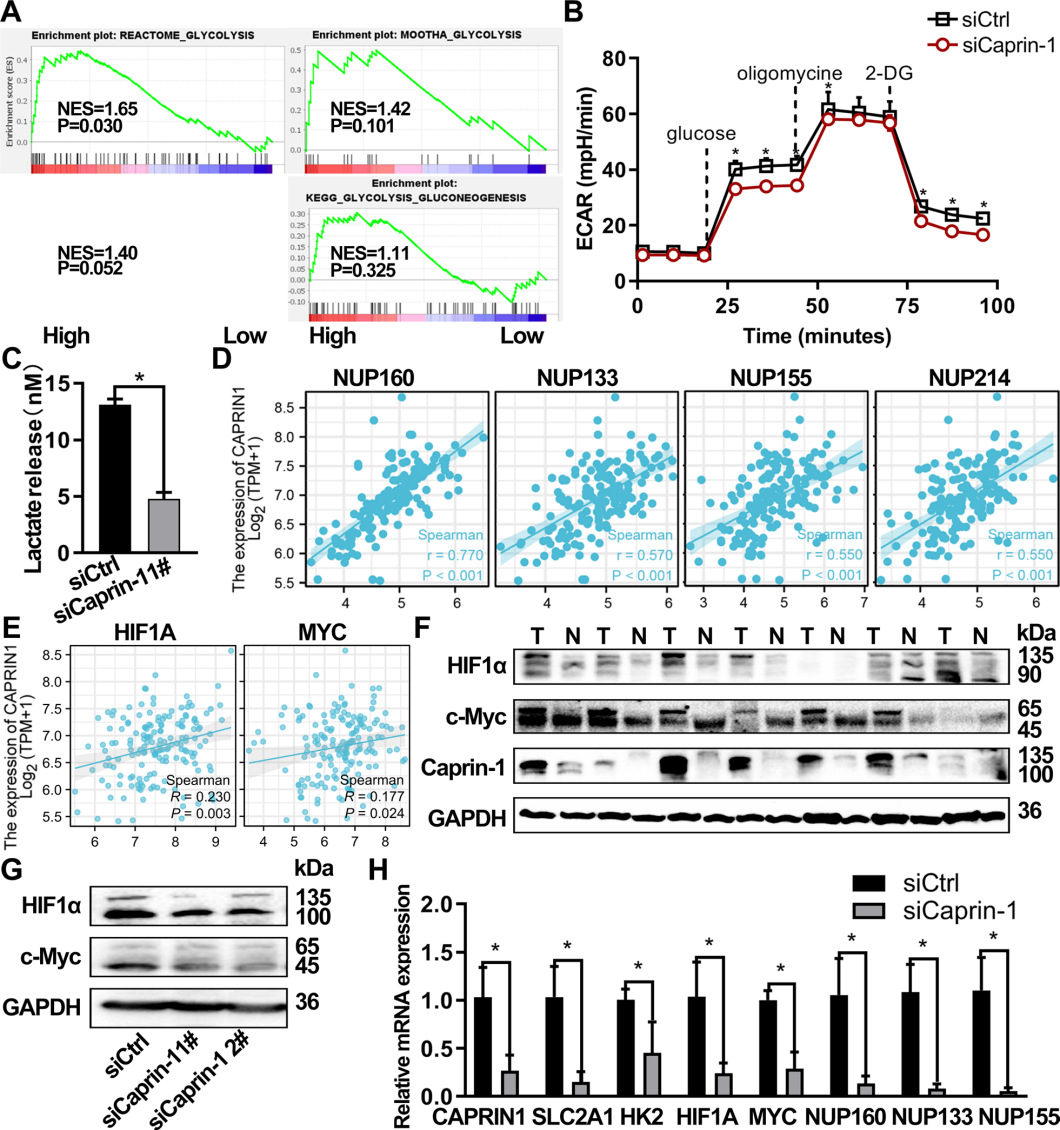
Knockdown of Caprin-1 inhibited glycolytic metabolism through the down-regulation of SLC2A1, HK2, HIF1A, MYC, NUP160, NUP133, and NUP155. **A** Gene-set enrichment analysis (GSEA) of the interrogated glycolysis signatures between the high and low CAPRIN1 groups in the database (GSE69925); NES, normalized enrichment score. **B** ECAR were measured in Eca109 cells 48 h after Caprin-1 knockdown using the Seahorse XF24 extracellular flux analyzer. **C** Lactate production was assessed in Eca109 cells after 48 h of siRNA transfection. **D** Correlation between CAPRIN1 and the top four leading-edge genes contributing to GSEA in the TCGA database. **E** Correlation between CAPRIN1 and glycolysis genes (HIF1A and MYC) in the TCGA database. **F** The protein expression level of HIF1α, c-Myc, and Caprin-1 in 7 paired ESCC tissues was examined using Western blot. T, tumor; N, adjacent normal tissues. **G** The expression patterns of HIF1α and c-Myc in Eca109 cells after silencing Caprin-1. **H** Changes in mRNA levels were detected by real time PCR after the Caprin-1 1# knockdown of Eca109 cells

GSEA results showed that the four glycolysis gene signatures were enriched in samples with high Caprin-1 expression. REACTOME_GLYCOLYSIS was significantly enriched at nominal p-value < 0.05 (Fig. 4A). We also observed significantly decreased ECAR and lactate release in Caprin-1 knockdown cells than in negative controls (Fig. 4B, C). Caprin-1 was positively correlated with the expression of *NUP160* (r = 0.770, *P* < 0.001), *NUP133* (r = 0.570, *P* < 0.001), *NUP155* (r = 0.550, *P* < 0.001), and *NUP214* (r = 0.550, *P* < 0.001) in TCGA-ESCA samples (Fig. 4D). Meanwhile, Caprin-1 was positively correlated with the expression of *HIF1A* and *MYC* (*P* < 0.05) (Fig. 4E). Western blot further confirmed that HIF1α and c-Myc expression was elevated in ESCA tissues than in paracancerous and normal tissues, and in the same pattern with that of Caprin-1 (Fig. 4F). After silencing Caprin-1 in the ESCA cells, HIF1α and c-Myc expression decreased (Fig. 4G). Eca109 cells transfected with siCaprin-1 had significantly decreased mRNA expression of glycolysis related genes, *SLC2A1*, *HK2, HIF1A, MYC, NUP160, NUP133, and NUP155*, compared to those transfected with control siRNA (Fig. 4H).

### Relationship between Caprin-1 expression and glucose metabolic asymmetries

**Fig. 5:**
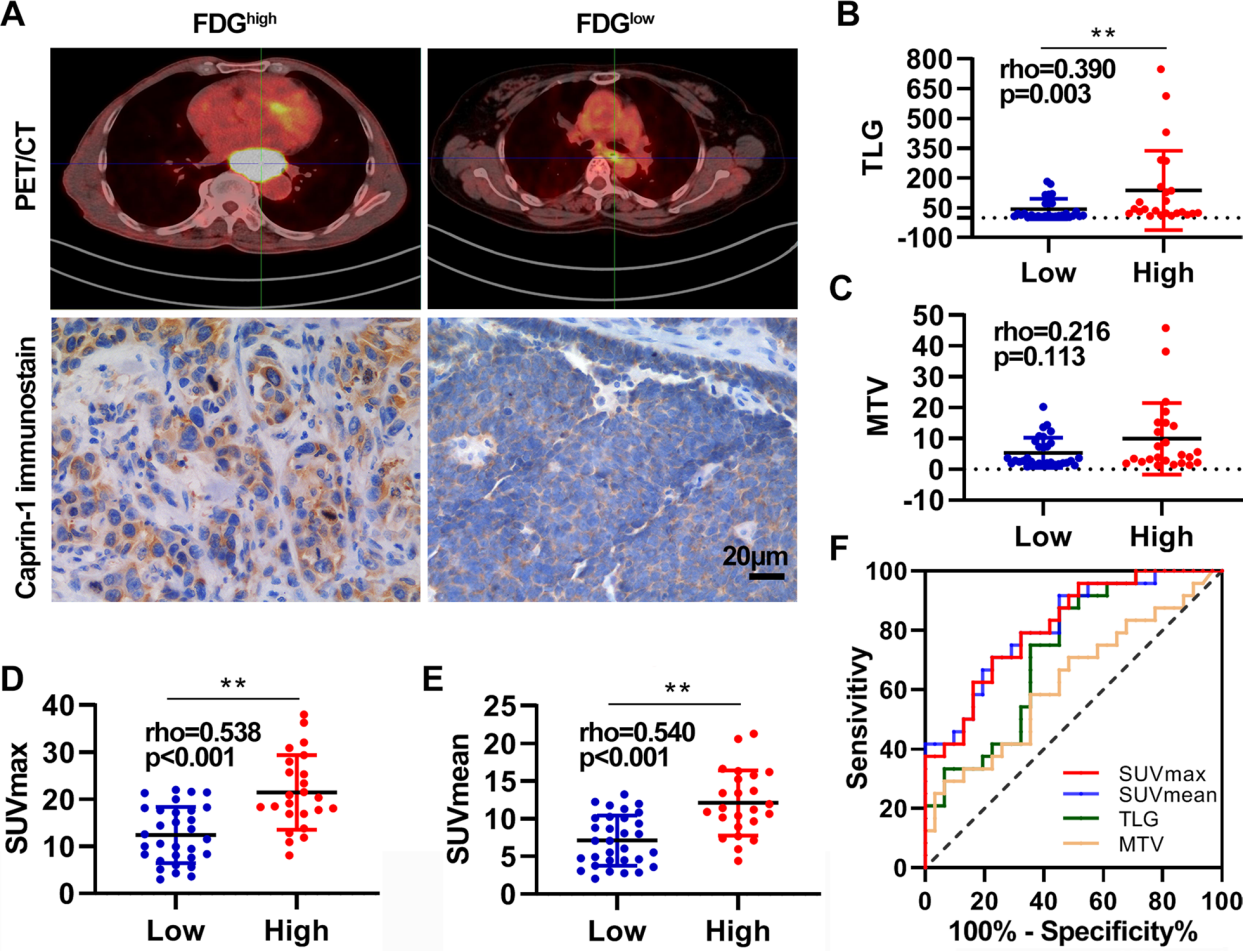
High expression of Caprin-1 was correlated with high glycolytic activity based on metabolic parameters in ^18^F-FDG PET imaging. **A** Representative images of PET/CT imaging and immunohistochemical staining for Caprin-1 in ESCC patients with high SUVmax (left) and low SUVmax (right). **B** The TLG, MTV **C**, SUVmax **D**, and SUVmean **E** showed a linear correlation with the protein levels of Caprin-1 with correlation coefficients of 0.390, 0.216, 0.538, and 0.540 respectively. **F** The determination of the cutoff value of SUVmax, SUVmean, TLG, and MTV by the receiver operating characteristics (ROC) curve

To further explore the role of Caprin-1 expression on glucose metabolism in ESCA, the potential association between ^18^F-FDG PET/CT metabolic parameters and Caprin-1 expression was assessed in 55 tumour samples. Two typical PET images and IHC staining for Caprin-1 of two case are shown in Fig. 5A. SUVmax and SUVmean were significantly different between groups according to Caprin-1 expression (Table 3). SUVmax, SUVmean, and TLG were positively correlated in a linear correlaion with IHC intensity levels of Caprin-1 (rho = 0.538, 0.540, and 0.390, respectively; *P* < 0.01), while the Caprin-1 intensity score showed no statistical correlation with MTV (Fig. 5B–E). Multivariate analysis results are shown in Table 4. Lymph node metastasis (*P* = 0.022) and SUVmax (*P* = 0.048) were strongly correlated with Caprin-1 expression. The area under the SUVmax, SUVmean, TLG, and MTV ROC curves was 0.813, 0.814, 0.727, and 0.626, respectively. Sensitivity of both SUVmax and SUVmean to predict Caprin-1 expression was 70.8% and their specificity was 77.4% (Fig. 5F). Therefore, SUVmax of 17.71 or SUVmax of 10.14 were suitable critical values for the prediction of Caprin-1 expression in ESCA.

**Table 3:** Comparison of PET metabolic parameter according to Caprin-1 expression

PET metabolic parameter	Caprin-1-Low (*n* = 31)	Caprin-1-High (*n* = 24)	*P*-value
SUV_max_ (mean ± SD)	12.43 ± 5.96	21.45 ± 7.92	0.002*
SUV_mean_ (mean ± SD)	7.11 ± 3.35	12.09 ± 4.33	0.005*
TLG(median, range)	13.85, 1.95–183.00	39.67, 8.88–151.90	0.120
MTV (median, range)	2.69, 0.81–20.22	4.26, 1.16–45.82	0.175

*P < 0.05

**Table 4:** Multivariate analysis of Caprin-1 expression status in patients with ESCA

parameter	OR	95% CI	*P*-value
Gender	0.683	0.1014–4.609	0.695
Age	1.726	0.404–7.379	0.461
Histological differentiation	2.646	0.547–12.789	0.226
Lymph node metastasis	0.142	0.027–0.757	0.022*
SUV_max_	0.043	0.002–1.046	0.048*
SUV_mean_	1.020	0.059–17.762	0.989
TLG	3.762	0.281–50.393	0.317
MTV	0.579	0.060–5.609	0.637

*P < 0.05

### Silencing Caprin-1 delayed ESCA progression by downregulating METTL3 and WTAP expression

**Fig. 6:**
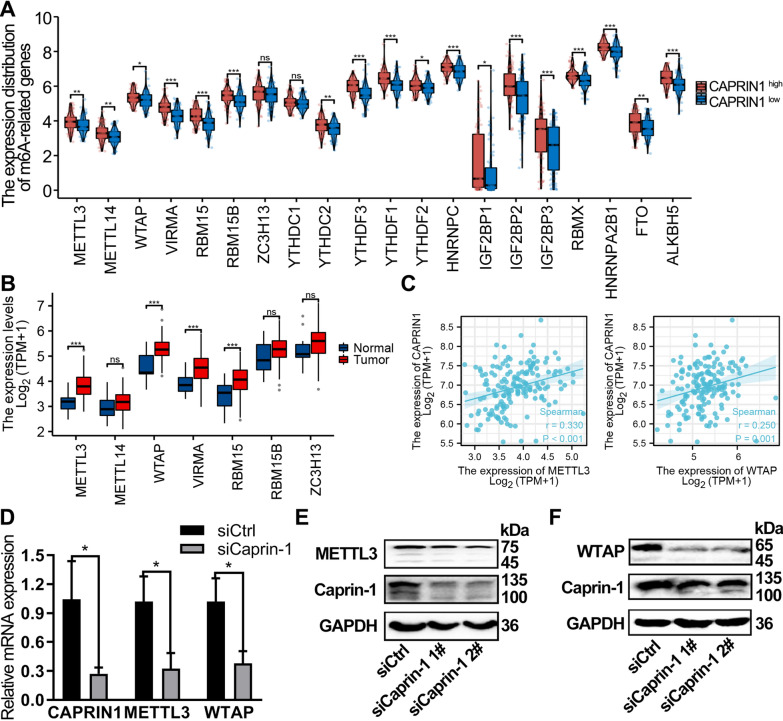
Caprin-1 expression was significantly correlated with WTAP and METTL3 in ESCA. **A** Comparison of m6A related genes in TCGA-ESCA tumors with high or low CAPRIN1 expression. **B** Expression pattern of m6A writers in ESCA tumor tissue and normal tissues as shown in the TCGA database. **C** The co-expression of CAPRIN1 with METTL3 and WTAP in ESCA from the TCGA database. **D** The expression patterns of METTL3 and WTAP in Eca109 cells after silencing Caprin-1 were determined by qRT-PCR and Western blot assay **E**, **F**

Compared with the CAPRIN1-low expression group (n = 81), the CAPRIN1-high expression group (n = 81) showed statistically significant correlation with all m6A-related genes except ZC3H13 and YTHDC1 in TCGA-ESCA (*P* < 0.05) (Fig. 6A). The mRNA expression of METTL3, WTAP, VIRMA and RBM15 increased in the ESCA samples (Fig. 6B). CAPRIN1 expression had a significantly positive correlation with m6A writers in ESCA, including METTL3 (r = 0.330, *P* < 0.001) and WTAP (r = 0.250, *P* = 0.001) (Fig. 6C). After silencing Caprin-1 in the ESCA cells, METTL3 and WTAP expression in ESCA cells decreased (Fig. 6D–E).

**Fig. 7:**
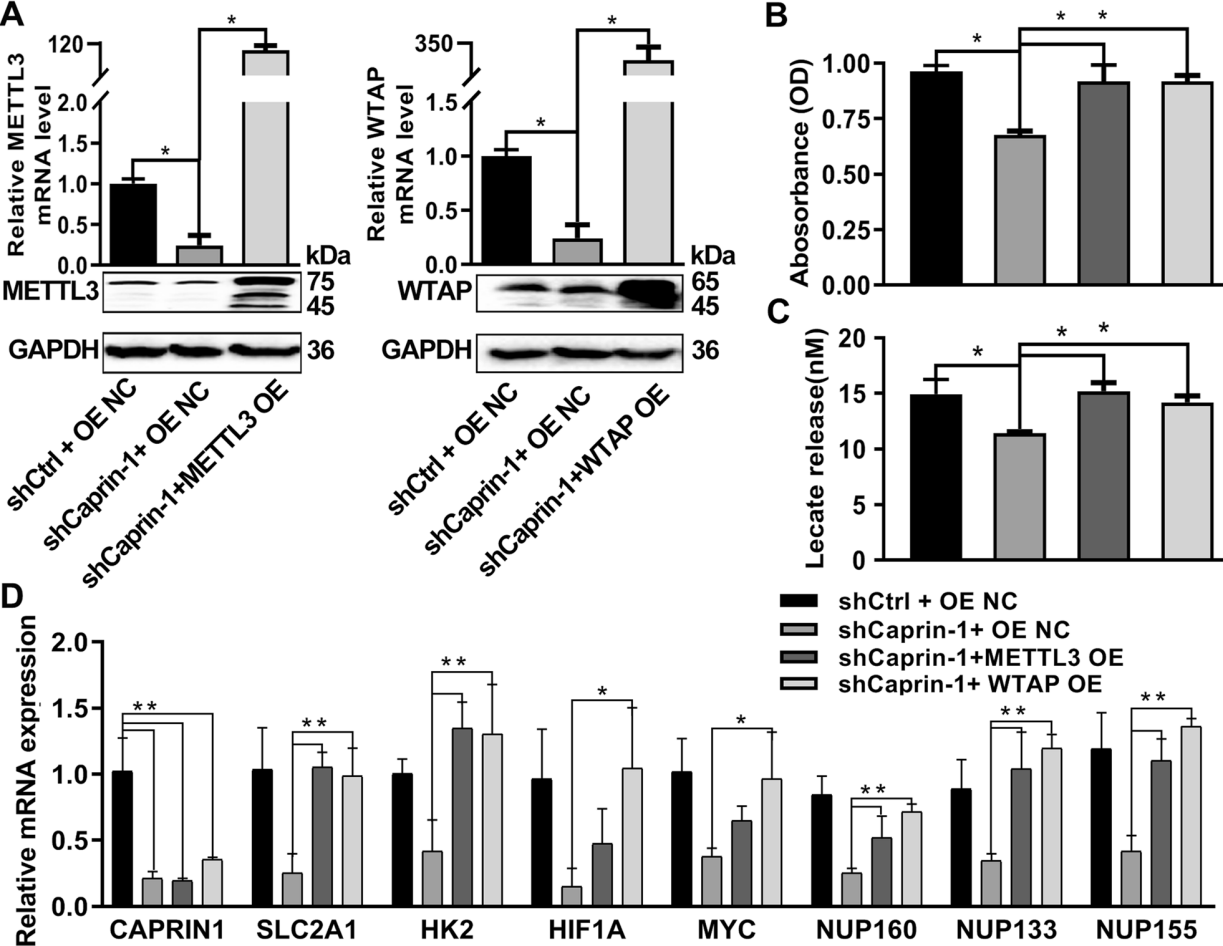
Overexpression of METTL3 and WTAP rescued the cell proliferation phenotype induced by silencing Caprin-1. **A** The mRNA and proteins of METTL3 and WTAP in Eca109 cells upon Caprin-1 silencing or combined with METTL3 and WTAP overexpression. **B** The cell viability and lactate release **C** of Eca109 cells upon Caprin-1 silencing or combined with METTL3 and WTAP overexpression. **D** qRT-PCR analysis was applied in Eca109 cells using indicated PCR primers

The expression of METTL3 and WTAP in ESCA cells was reverted by METTL3 and WTAP overexpression, compared to the shCaprin-1 group (Fig. 7A). Proliferation and lactate release was suppressed in ESCA cells upon Caprin-1 silencing; however, the restoration of METTL3 and WTAP partially reversed these effects in a significantly manner (*P* < 0.05, Fig. 7B and C). WTAP OE could rescue the Caprin-1 shRNA-induced decreased in mRNA expression of glycolysis related genes (*SLC2A1, HK2, HIF1A, MYC, NUP160, NUP133,* and *NUP155*); METTL3 OE could rescue the mRNA expression of *SLC2A1*, *HK2*, *NUP160*, *NUP133*, and *NUP155* (Fig. 7D). METTL3 or WTAP overexpression could reverse the inhibitory effect of Caprin-1 silencing on the proliferation and glycolysis of ESCA cells.

### Knockdown of Caprin-1 inhibited tumour growth in vivo

**Fig. 8:**
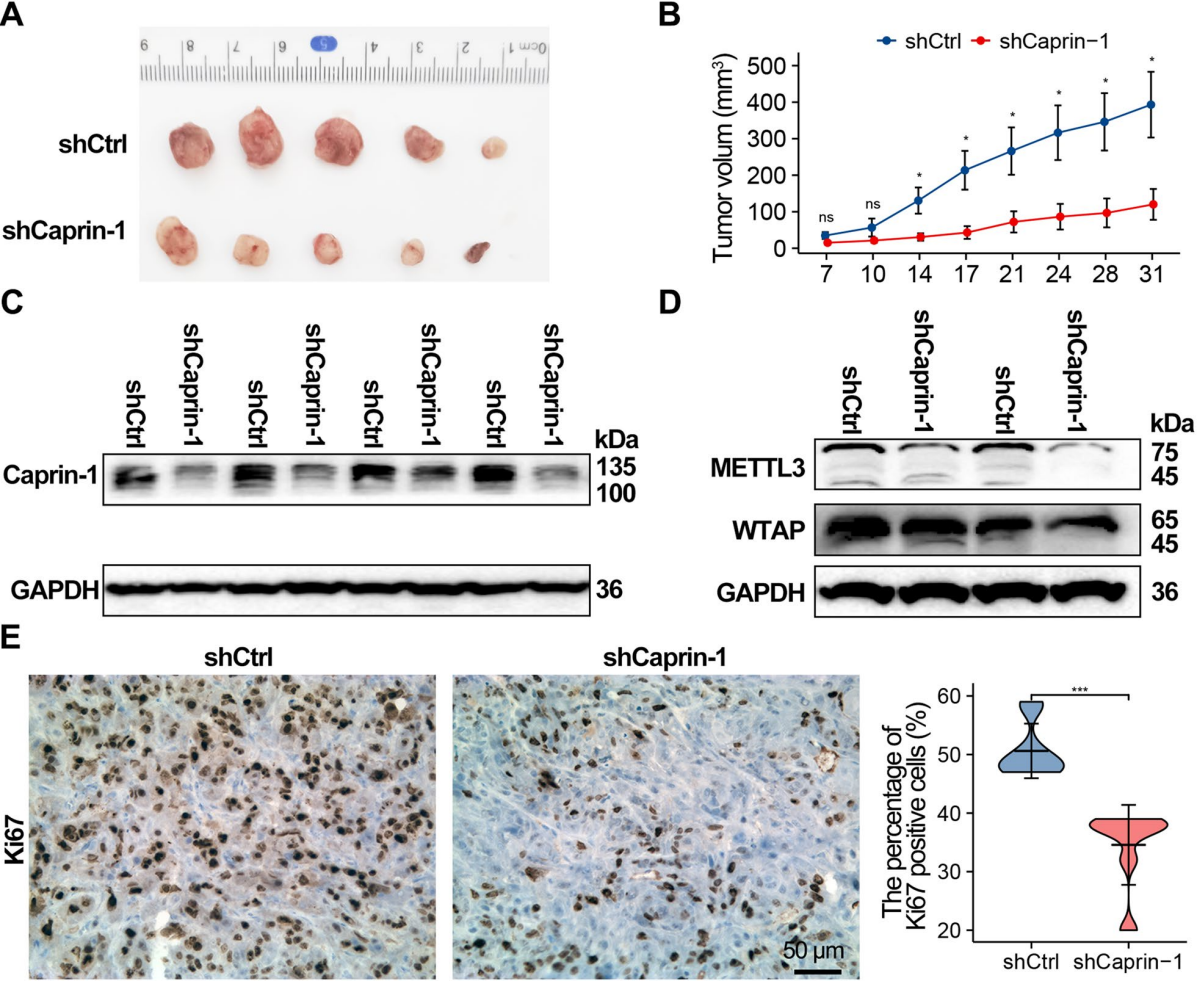
The growth of ESCA xenografts is inhibited by silencing Caprin-1. **A** Representative images of transplanted tumors in nude mice injected with Eca109 cells. **B** Tumour growth curve of shCtrl and shCaprin-1 group (n = 5). **C**, **D** Western blot analysis of Caprin-1, METTL3, and WTAP expression in tumor tissues. **E** IHC analysis of Ki67 in xenografs. The histogram indicates the Ki67 positive cells. *P < 0.05, ***P < 0.001

Finally, to further investigate the impact of Caprin-1 on tumor growth in vivo, the subcutaneous xenograft tumor model was established in nude mice using Caprin-1 knockdown (shCaprin-1) or the control cells (shCtrl). As expected, tumor grew dramatically slower in shCaprin-1 group compared with the control group (*P* < 0.05) (Fig. 8A and B). Further studies showed that knockdown of Caprin-1 in vivo inhibited protein expression of Caprin-1, METTL3, and WTAP (Fig. 8C and D). Additionally, IHC analyses showed that a significant decrease in the positive rate of Ki67 in Caprin-1 knockdown group (Fig. 8E). Overall, these results demonstrated that Caprin-1 silencing could inhibit tumor growth in vivo.

## Discussion

Caprin-1 is up-regulated in different cancer types [33], including lung [15, 34], prostate [16], breast [17], gastric [35], liver [18, 36] and colon cancer [37]. No studies have reported the underlying biological function of Caprin-1 in ESCA. Herein, we found that CAPRIN1 mRNA expression was up-regulated and associated with poor prognosis and good diagnostic accuracy in ESCA. Increased levels of Caprin-1 mRNA and protein expression were associated with lymph node metastasis and PET metabolic parameters in ESCA patients. PET parameters (SUVmax and SUVmean) might be suitable predictors of Caprin-1 expression in ESCA patients. Additionally, CAPRIN1 might promote ESCA progression through glycolytic reprogramming and regulation of m6A writers (METTL3 and WTAP). Therefore, we identified Caprin-1 as a potential biomarker that modulates glucose metabolism and m6A writers in ESCA.

The absence of Caprin-1 leads to cancer cell proliferation, migration, and invasion defects [15, 17] and immune cells proliferation [20]. In this study, the GO enrichment and KEGG pathway analysis CAPRIN1 might be related to immune-related terms, protein binding processes, and metabolic pathways. Meanwhile, further verification experiments with IHC confirmed that Caprin-1 expression was significantly associated with Ki-67 expression (strictly associated with cell proliferation) in ESCC patients. The in vitro and in vivo results revealed that knockdown of Caprin-1 inhibited ESCC cell proliferation. While the clinical outcomes and pathophysiology of ESCA and OA seems to be different, Caprin-1 expression displayed different OS patterns in these cancers. The Warburg effect is associated with malignant features of cancers, and is measured by high glycolytic rates, increased lactate release, and several intermediates and enzymes for rapidly proliferating cells [38]. Recent studies have revealed that Caprin-1 expression is functionally required for glutamine metabolism [39], which also involves cell proliferation and metastasis in various cancers [40]. Kim et al. [12] showed that CAPRIN1 controls RNA processing and translation, which regulates human and mouse metabolism [41]. However, whether Caprin-1 expression participates in the regulation of glucose metabolism remains unclear.

Our results demonstrated that the glycolysis pathway was significantly enriched in response to high CAPRIN1 expression in ESCA patients. Consistently, Caprin-1 knockdown inhibited glycolysis, followed by down-regulating the commonly glycolytic genes (*SLC2A1*, *HK2*, *HIF1A*, and *MYC*) and glycolytic genes screened from the PPI network (nucleoporins *NUP160*, *NUP155*, and *NUP133*) in ESCA cells. Given that Nup160, Nup133 [42] and Caprin-1 [14] play key roles in mRNA export, the links among them warrants further investigation. The fact that CAPRIN1 can bind directly and selectively to c-Myc and HIF-α has been reported previously [43]. MYC activity interference has been implicated in many malignant phenotypes of human cancers, including the Warburg effect [44]. It has been proven that HIFα acts as a major mediator of transcription in response to hypoxia, and provokes cell proliferation and the Warburg effect [45]. Surov et al.[46] reported a significant correlation of HIF-α expression and Ki-67 levels with PET parameters in head and neck squamous cell carcinoma. The results of the present study corroborated those of previous reports and showed that Caprin-1 expression had a significantly positive association with ^18^F-FDG PET parameters, which can reflect the glucose metabolism and growth patterns of tumor cells in ESCA patients. Additionally, ^18^F-FDG uptake may have a role in predicting Caprin-1 expression. These results suggest that Caprin-1 promotes ESCA tumorigenesis by inducing the Warburg effect.

Caprin-1 plays critical roles in m6A modification and regulates m6A disruption in eukaryotic RNA [19, 47]. Using RNA chemical proteomics, Arguello et al. [47] revealed that the CAPRIN1, G3BP1/2, RBM42, and USP10 are repelled by the m6A modification. Our results showed a significantly positive correlation of CAPRIN1 with most m6A regulators in ESCA. Previous studies in 25 cell lines also showed that CAPRIN1 knockdown selectively modulated m6A sites within the coding regions through interactions with m6A writers (METTL3 and METTL14)[19]. Our results showed that the expression of m6A writers (METTL3, WTAP, VIRMA and RBM15) was significantly elevated in clinical ESCA tissues, whereas expression of METTL14, RBM15B, and ZC3H13 was not significantly changed. Among these, METTL3 promotes aerobic glycolysis and ESCC tumour development [23]. Interfering with METTL3 expression can reduce the level of m6A modification of HIF1α and inhibit the metabolic reprogramming of colorectal and liver cancer cells [48, 49]. The elevated expressions of WTAP and VIRMA genes were strongly correlated with poor prognosis in ESCA patients, augmented levels of WTAP were associated with poor OS of EA [50], whereas WTAP promoted the proliferation and migration of ESCC [51]. Both METTL3 and WTAP accelerates the Warburg effect of gastric cancer [52, 53]. We found that there were positive correlations among METTL3, WTAP, and CAPRIN1 in TCGA-ESCA tissues. Besides, CAPRIN1 knockdown inhibits ESCC cell proliferation and tumour growth and decreased the expression of METTL3 and WTAP. Therefore, we speculated that Caprin-1 might affect ESCA progression by affecting METTL3 and WTAP. To further validate this speculation, we conducted a functional rescue experiment. As expected, our validation experiments confirmed that Caprin-1 knockdown significantly inhibited the growth rate and lactate production of ESCA cells, which could be rescued by METTL3 and WTAP overexpression. The underlying mechanism is that Caprin-1 knockdown inhibited cell proliferation and metabolism through inhibiting the mRNA expression of glycolysis related genes (*SLC2A1*, *HK2*, and so on), which could also be rescued by METTL3 or WTAP overexpression. In keeping with our findings, previous studies have reported that METTL3 directly interacted and stabilized with *HK2* and *SLC2A1*, then further activated the glycolysis pathway [53, 54]. Moreover, WTAP was found to be closely associated with *HIF-1α* and *MYC* [55, 56]. Thus, we conclude that Caprin-1 could affect the ESCA cell proliferation by up-regulating METTL3 and WTAP expression.

## Conclusion

In conclusion, our preliminary findings showed that Caprin-1 was highly expressed in cancer tissues and significantly associated with prognosis in diverse cancer types. Caprin-1 might promote ESCA progression by regulating glycolysis reprogramming and m6A writers including METTL3 and WTAP. The Warburg effect, m6A RNA methylation, and the interaction between them are crucial for tumor progression [57]. Although in vitro studies have been conducted to explore the potential function of Caprin-1, the underlying mechanisms of signaling pathways in ESCC has not been fully elucidated, thus further investigation is required to confirm the role of Caprin-1 in ESCA growth and progression in patients, and its relationship with glycolysis and m6A methylation in the tumor microenvironment.

## Supplementary Information

**Additional file 1: Figure S1.** Bioinformatic analysis of CAPRIN1 mRNA expression. (A) Comparison of CAPRIN1 expression among age (> 60 and ≤ 60) and race (Asian, White, and Black or African American) (B) groups in the TCGA-ESCA and ESCC database. (C) PPI analysis of the CAPRIN1 correlated genes. (D) Enriched BP, CC, and MF GO terms in the differentially expressed genes in the LinkedOmics database.

## Data Availability

Publicly available datasets were analyzed in this study. The datasets used and/or analyzed during the current study are available from the corresponding author on reasonable request.

## Abbreviations

Caprin-1: Cytoplasmic activation/proliferation-associated protein-1
ESCA: Esophageal carcinoma
PET/CT: Positron emission tomography/computed tomography
^18^F-FDG: ^18^F-fludeoxyglucose
ESCC: Esophageal squamous cell carcinoma
EA: Esophageal adenocarcinoma
m6A: N6-methylandenosin
TCGA: The Cancer Genome Atlas
GEO: Gene Expression Omnibus
GSEA: Gene-set enrichment analysis
METTL3: Methyltransferase-like 3
WTAP: Wilms’ tumor 1-associating protein
METTL14: Methyltransferase-like 14
SUVmax: Maximum Standardized Uptake Value
SUVmean: Mean Standardized Uptake Value
MTV: Metabolic tumor volume
TLG: Total lesion glycolysis
ECARs: Extracellular acidification rates

## References

[1] Frankell AM, Jammula S, Li X, Contino G, Killcoyne S, Abbas S, Perner J, Bower L, Devonshire G, Ococks E (2019). The landscape of selection in 551 esophageal adenocarcinomas defines genomic biomarkers for the clinic. Nat Genet.

[2] Essadi I, Lalya I, Mansouri H (2015). Esophageal carcinoma. N Engl J Med.

[3] Nam AS, Chaligne R, Landau DA (2021). Integrating genetic and non-genetic determinants of cancer evolution by single-cell multi-omics. Nat Rev Genet.

[4] Sanchez-Vega F, Mina M, Armenia J, Chatila WK, Mariamidze A (2018). Oncogenic signaling pathways in the cancer genome Atlas. Cell.

[5] Thakur C, Chen F (2019). Connections between metabolism and epigenetics in cancers. Semin Cancer Biol.

[6] Flamen P, Lerut A, Cutsem EV, Wever WD, Mortelmans L (2000). Utility of positron emission tomography for the staging of patients with potentially operable esophageal carcinoma. J Clin Oncol.

[7] Hofheinz F, Li Y, Steffen IG, Lin Q, Lili C, Hua W, van den Hoff J, Zschaeck S (2019). Confirmation of the prognostic value of pretherapeutic tumor SUR and MTV in patients with esophageal squamous cell carcinoma. Eur J Nucl Med Mol Imaging.

[8] Wang Y, Zhao N, Wu Z, Pan N, Shen X, Liu T, Wei F, You J, Xu W, Ren X (2020). New insight on the correlation of metabolic status on 18F-FDG PET/CT with immune marker expression in patients with non-small cell lung cancer. Eur J Nucl Med Mol Imaging.

[9] Kaira K, Endo M, Abe M, Nakagawa K, Ohde Y, Okumura T, Takahashi T, Murakami H, Tsuya A, Nakamura Y, Naito T, Hayashi I, Serizawa M, Koh Y, Hanaoka H, Tominaga H, Oriuchi N, Kondo H, Nakajima T, Yamamoto N (2010). Biologic correlation of 2-[18F]-fluoro-2-deoxy-D-glucose uptake on positron emission tomography in thymic epithelial tumors. J Clin Oncol.

[10] Zhou L, Yuan L, Gao Y, Liu X, Dai Q, Yang J, Pei Z (2021). Nucleophosmin 1 overexpression correlates with 18F-FDG PET/CT metabolic parameters and improves diagnostic accuracy in patients with lung adenocarcinoma. Eur J Nucl Med Mol Imaging.

[11] Liu X, Yuan L, Gao Y, Zhou L, Yang J, Pei Z (2020). Overexpression of METTL3 associated with the metabolic status on 18F-FDG PET/CT in patients with Esophageal Carcinoma. J Cancer.

[12] Kim TH, Tsang B, Vernon RM, Sonenberg N, Kay LE, Forman-Kay JD (2019). Phospho-dependent phase separation of FMRP and CAPRIN1 recapitulates regulation of translation and deadenylation. Science (American Association for the Advancement of Science).

[13] Shiina N, Yamaguchi K, Tokunaga M (2010). RNG105 deficiency impairs the dendritic localization of mRNAs for Na+/K+ ATPase subunit isoforms and leads to the degeneration of neuronal networks. J Neurosci.

[14] Nakayama K, Ohashi R, Shinoda Y, Yamazaki M, Abe M, Fujikawa A, Shigenobu S, Futatsugi A, Noda M, Mikoshiba K (2017). RNG105/caprin1, an RNA granule protein for dendritic mRNA localization, is essential for long-term memory formation. Elife.

[15] Sabile AA, Arlt MJE, Muff R, Husmann K, Hess D, Bertz J, Langsam B, Aemisegger C, Ziegler U, Born W, Fuchs B (2013). Caprin-1, a novel Cyr61-interacting protein, promotes osteosarcoma tumor growth and lung metastasis in mice. Biochim Biophys acta Mol Basis Dis.

[16] Shi Q, Zhu Y, Ma J, Chang K, Ding D, Bai Y, Gao K, Zhang P, Mo R, Feng K (2019). Prostate Cancer-associated SPOP mutations enhance cancer cell survival and docetaxel resistance by upregulating Caprin1-dependent stress granule assembly. Mol Cancer.

[17] Gong B, Hu H, Chen J, Cao S, Yu J, Xue J, Chen F, Cai Y, He H, Zhang L (2013). Caprin-1 is a novel microRNA-223 target for regulating the proliferation and invasion of human breast cancer cells. Biomed Pharmacother.

[18] Guo XM, Zhu FF, Pan LW, Chen JL, Lai JC, Wu HX, Shu JC (2020). Caprin-1 promotes HepG2 cell proliferation, invasion and migration and is associated with poor prognosis in patients with liver cancer. Oncol Lett.

[19] An S, Huang W, Huang X, Cun Y, Cheng W, Sun X, Ren Z, Chen Y, Chen W, Wang J (2020). Integrative network analysis identifies cell-specific trans regulators of m6A. Nucleic Acids Res.

[20] Wang B, David MD, Schrader JW (2005). Absence of Caprin-1 results in defects in cellular proliferation. J Immunol.

[21] Li Y, Xiao J, Bai J, Tian Y, Qu Y, Chen X, Wang Q, Li X, Zhang Y, Xu J (2019). Molecular characterization and clinical relevance of m6A regulators across 33 cancer types. Mol Cancer.

[22] Ping X, Sun B, Wang L, Xiao W, Yang X, Wang W, Adhikari S, Shi Y, Lv Y, Chen Y (2014). Mammalian WTAP is a regulatory subunit of the RNA N6-methyladenosine methyltransferase. Cell Res.

[23] Wang W, Shao F, Yang X, Wang J, Zhu R, Yang Y, Zhao G, Guo D, Sun Y, Wang J (2021). METTL3 promotes tumour development by decreasing APC expression mediated by APC mRNA N6-methyladenosine-dependent YTHDF binding. Nat Commun.

[24] Barrett T, Troup DB, Wilhite SE, Ledoux P, Soboleva A (2011). NCBI GEO: archive for functional genomics data sets - Update. Nucleic Acids Res.

[25] Gao Y, Li F, Zhou H, Yang Y, Wu R, Chen Y, Li W, Li Y, Xu X, Ke C, Pei Z (2017). Down-regulation of MRPS23 inhibits rat breast cancer proliferation and metastasis. Oncotarget.

[26] Zhu M, Gong Z, Wu Q, Shi X, Su Q, Zhang Y (2020). Sanguinarine suppresses migration and metastasis in colorectal carcinoma associated with the inversion of EMT through the Wnt/β-catenin signaling. Clin Transl Med.

[27] Li T, Fan J, Wang B, Traugh N, Chen Q, Liu JS, Li B, Liu XS (2017). TIMER: a web server for comprehensive analysis of tumor-infiltrating immune cells. Cancer Res.

[28] Nagy D, Munkácsy G, Gyrffy B (2021). Pancancer survival analysis of cancer hallmark genes. Sci Rep.

[29] Vasaikar SV, Peter S, Jing W, Bing Z (2018). LinkedOmics: analyzing multi-omics data within and across 32 cancer types. Nucleic Acids Res.

[30] Pei Z, Zeng J, Gao Y, Li F, Li W, Zhou H, Yang YI, Wu R, Chen Y, Liu J (2016). Oxymatrine inhibits the proliferation of CaSki cells via downregulating HPV16E7 expression. Oncol Rep.

[31] Zhu M, Gong Z, Wu Q, Su Q, Yang T, Yu R, Xu R, Zhang Y (2020). Homoharringtonine suppresses tumor proliferation and migration by regulating EphB4-mediated β-catenin loss in hepatocellular carcinoma. Cell Death Dis.

[32] Zhang R, Shen M, Wu C, Chen Y, Lu J, Li J, Zhao L, Meng H, Zhou X, Huang G (2020). HDAC8-dependent deacetylation of PKM2 directs nuclear localization and glycolysis to promote proliferation in hepatocellular carcinoma. Cell Death Dis.

[33] Yang Z, Qing H, Gui H, Luo J, Dai L, Wang B (2019). Role of caprin-1 in carcinogenesis (Review). Oncol Lett.

[34] Liu X, Xiang D, Xu C, Chai R (2021). EIF3m promotes the malignant phenotype of lung adenocarcinoma by the up-regulation of oncogene CAPRIN1. Am J Cancer Res.

[35] Lu Q, Chen Y, Sun D, Wang S, Ding K, Liu M, Zhang Y, Miao Y, Liu H, Zhou F (2019). MicroRNA-181a functions as an oncogene in gastric cancer by targeting Caprin-1. Front Pharmacol.

[36] Tan N, Dai L, Liu X, Pan G, Chen H, Huang J, Xu Q (2017). Upregulation of caprin1 expression is associated with poor prognosis in hepatocellular carcinoma. Pathol Res Pract.

[37] Teng Y, Ren Y, Hu X, Mu J, Samykutty A, Zhuang X, Deng Z, Kumar A, Zhang L, Merchant ML (2017). MVP-mediated exosomal sorting of miR-193a promotes colon cancer progression. Nat Commun.

[38] Heiden MGV, Cantley LC, Thompson CB (2009). Understanding the warburg effect: the metabolic requirements of cell proliferation. Science.

[39] Wang R, Cao L, Thorne RF, Zhang XD, Li J, Shao F, Zhang L, Wu M (2021). LncRNA GIRGL drives CAPRIN1-mediated phase separation to suppress glutaminase-1 translation under glutamine deprivation. Sci Adv.

[40] Altman BJ, Stine ZE, Dang CV (2016). From Krebs to clinic: glutamine metabolism to cancer therapy. Nat Rev Cancer.

[41] Leboucher A, Pisani DF, Martinez-Gili L, Chilloux J, Bermudez-Martin P, Van Dijck A, Ganief T, Macek B, Becker JAJ, Le Merrer J (2019). The translational regulator FMRP controls lipid and glucose metabolism in mice and humans. Mol Metab.

[42] Vasu S, Shah S, Orjalo A, Park M, Fischer WH, Forbes DJ (2001). Novel vertebrate nucleoporins Nup133 and Nup160 play a role in mRNA export. J Cell Biol.

[43] Lee YZ, Guo HC, Zhao GH, Yang CW, Chang HY, Yang RB, Chen L, Lee SJ (2020). Tylophorine-based compounds are therapeutic in rheumatoid arthritis by targeting the caprin-1 ribonucleoprotein complex and inhibiting expression of associated c-Myc and HIF-1alpha. Pharmacol Res.

[44] Schaafsma E, Zhao Y, Zhang L, Li Y, Cheng C (2021). MYC activity inference captures diverse mechanisms of aberrant MYC pathway activation in human cancers. Mol Cancer Res.

[45] Jia D, Lu M, Jung KH, Park JH, Yu L, Onuchic JN, Kaipparettu BA, Levine H (2019). Elucidating cancer metabolic plasticity by coupling gene regulation with metabolic pathways. Proc Natl Acad Sci.

[46] Surov A, Meyer HJ, Hhn AK, Sabri O, Purz S (2019). Combined metabolo-volumetric parameters of 18 F-FDG-PET and MRI can predict tumor cellularity, Ki67 level and expression of HIF 1alpha in head and neck squamous cell carcinoma: a pilot study. Transl Oncol.

[47] Arguello AE, DeLiberto AN, Kleiner RE (2017). RNA chemical proteomics reveals the n6-methyladenosine (m6A)-regulated protein–RNA interactome. J Am Chem Soc.

[48] Yang N, Wang T, Li Q, Han F, Wang Z, Zhu R, Zhou J (2021). HBXIP drives metabolic reprogramming in hepatocellular carcinoma cells via METTL3-mediated m6A modification of HIF-1α. J Cell Physiol.

[49] Yang Z, Quan Y, Chen Y, Huang Y, Huang R, Yu W, Wu D, Ye M, Min Z, Yu B (2021). Knockdown of RNA N6-methyladenosine methyltransferase METTL3 represses Warburg effect in colorectal cancer via regulating HIF-1α. Signal Transduct Target Ther.

[50] Zhao H, Xu Y, Xie Y, Zhang L, Gao M, Li S, Wang F (2021). m6A regulators is differently expressed and correlated with immune response of esophageal cancer. Front Cell Dev Biol.

[51] Luo G, Qi Y, Lei Z, Shen X, Chen M, Du L, Wu C, Bo J, Wang S, Zhao J, Yi X (2022). A potential biomarker of esophageal squamous cell carcinoma WTAP promotes the proliferation and migration of ESCC. Pathol - Res Pract.

[52] Yu H, Zhao K, Zeng H, Li Z, Chen K, Zhang Z, Li E, Wu Z (2021). N6-methyladenosine (m6A) methyltransferase WTAP accelerates the Warburg effect of gastric cancer through regulating HK2 stability. Biomed Pharmacother.

[53] Shen C, Xuan B, Yan T, Ma Y, Xu P, Tian X, Zhang X, Cao Y, Ma D, Zhu X (2020). m6A-dependent glycolysis enhances colorectal cancer progression. Mol Cancer.

[54] Wang Q, Chen C, Ding Q, Zhao Y, Wang Z, Chen J, Jiang Z, Zhang Y, Xu G, Zhang J (2020). METTL3-mediated m6A modification of HDGF mRNA promotes gastric cancer progression and has prognostic significance. Gut.

[55] Naren D, Yan T, Gong Y, Huang J, Zhang D, Sang L, Zheng X, Li Y (2021). High Wilms’ tumor 1 associating protein expression predicts poor prognosis in acute myeloid leukemia and regulates m6A methylation of MYC mRNA. J Cancer Res Clin Oncol.

[56] Lyu Y, Zhang Y, Wang Y, Luo Y, Ding H, Li P, Ni G (2022). HIF-1α regulated WTAP overexpression promoting the Warburg effect of ovarian cancer by m6A-dependent manner. J Immunol Res.

[57] Wang H, Hu X, Huang M, Liu J, Gu Y, Ma L, Zhou Q, Cao X (2019). Mettl3-mediated mRNA m6A methylation promotes dendritic cell activation. Nat Commun.

