# Supplementary figures and images for: Caprin-1 plays a role in cell proliferation and Warburg metabolism of esophageal carcinoma by regulating METTL3 and WTAP
Source: J Transl Med. 2023 Feb 28;21:159. doi: 10.1186/s12967-023-04001-0 (PMC9976378; doi:10.1186/s12967-023-04001-0)

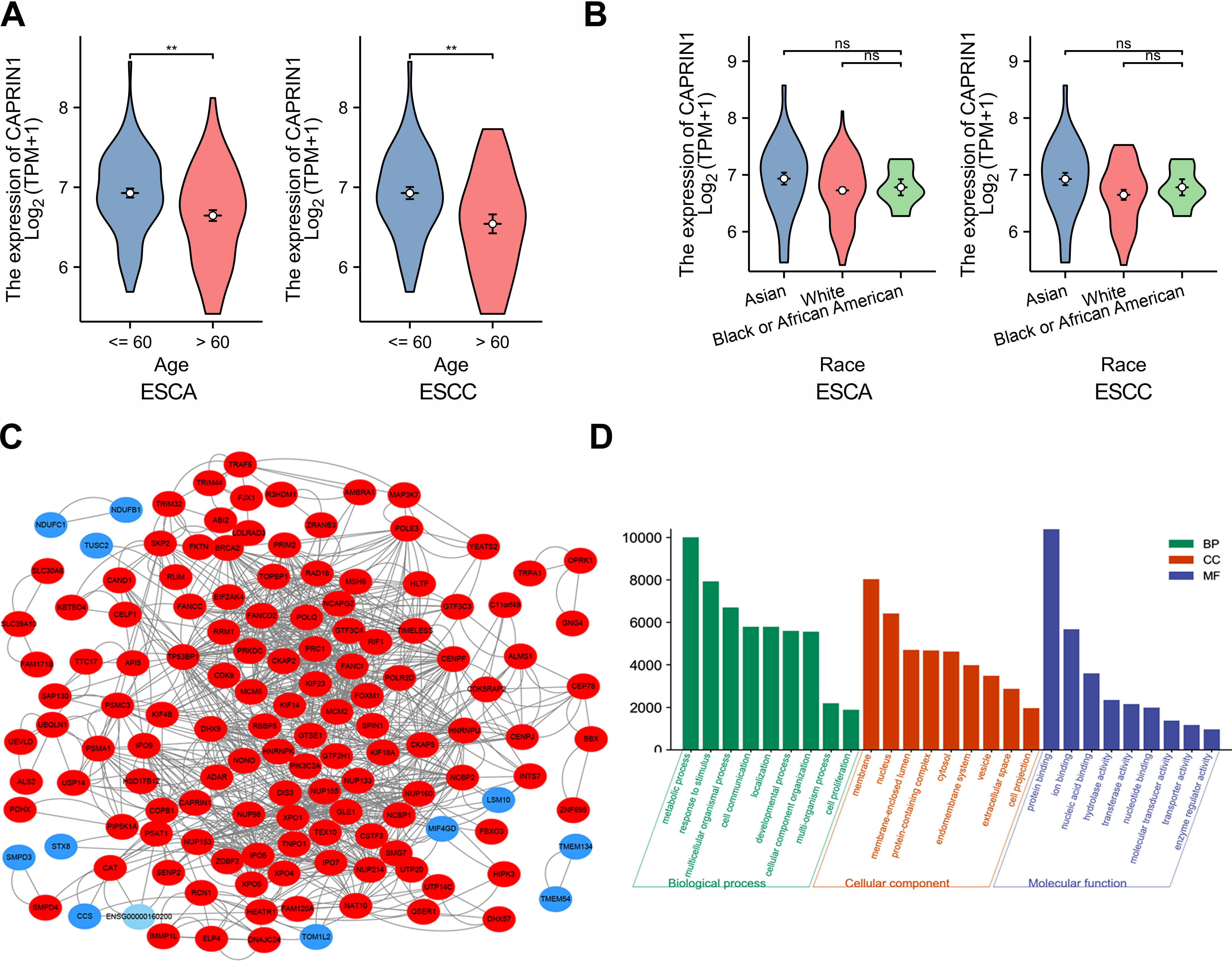

Supplement: Supplementary file 1 — Additional file 1: Figure S1. Bioinformatic analysis of CAPRIN1 mRNA expression. (A) Comparison of CAPRIN1 expression among age (> 60 and ≤ 60) and race (Asian, White, and Black or African American) (B) groups in the TCGA-ESCA and ESCC database. (C) PPI analysis of the CAPRIN1 correlated genes. (D) Enriched BP, CC, and MF GO terms in the differentially expressed genes in the LinkedOmics database. [file 12967_2023_4001_MOESM1_ESM.tif]
